# Benchmarking network for clinical and humanistic outcomes in diabetes (BENCH-D) study: protocol, tools, and population

**DOI:** 10.1186/2193-1801-3-83

**Published:** 2014-02-12

**Authors:** Antonio Nicolucci, Maria C Rossi, Fabio Pellegrini, Giuseppe Lucisano, Basilio Pintaudi, Sandro Gentile, Giampiero Marra, Soren E Skovlund, Giacomo Vespasiani, on behalf of the BENCH-D Study Group

**Affiliations:** Department of Clinical Pharmacology and Epidemiology, Mario Negri Sud Foundation, Via Nazionale, 66030S Maria Imbaro, CH Italy; Department of Clinical and Experimental Medicine, Second University of Naples, Naples, Italy; Novo Nordisk SpA, Rome, Italy; Skovlund SE, Novo Nordisk A/S, Bagsværd, Denmark; Diabetes Unit, Madonna del Soccorso Hospital, San Benedetto del Tronto, AP Italy

**Keywords:** Type 2 diabetes, Quality of care, Benchmarking, Patient-centered outcomes

## Abstract

**Background:**

In the context of the DAWN-2 initiatives, the BENCH-D Study aims to test a model of regional benchmarking to improve not only the quality of diabetes care, but also patient-centred outcomes.

**Methods/Design:**

As part of the AMD-Annals quality improvement program, 32 diabetes clinics in 4 Italian regions extracted clinical data from electronic databases for measuring process and outcome quality indicators. A random sample of patients with type 2 diabetes filled in a questionnaire including validated instruments to assess patient-centred indicators: SF-12 Health Survey, WHO-5 Well-Being Index, Diabetes Empowerment Scale, Problem Areas in Diabetes, Health Care Climate Questionnaire, Patients Assessment of Chronic Illness Care, Barriers to Medications, Patient Support, Diabetes Self-care Activities, and Global Satisfaction for Diabetes Treatment. Data were discussed with participants in regional meetings. Main problems, obstacles and solutions were identified through a standardized process, and a regional mandate was produced to drive the priority actions. Overall, clinical indicators on 78,854 patients have been measured; additionally, 2,390 patients filled-in the questionnaire. The regional mandates were officially launched in March 2012. Clinical and patient-centred indicators will be evaluated again after 18 months. A final assessment of clinical indicators will take place after 30 months.

**Discussion:**

In the context of the BENCH-D study, a set of instruments has been validated to measure patient well-being and satisfaction with the care. In the four regional meetings, different priorities were identified, reflecting different organizational resources of the different areas. In all the regions, a major challenge was represented by the need of skills and instruments to address psychosocial issues of people with diabetes. The BENCH-D study allows a field testing of benchmarking activities focused on clinical and patient-centred indicators.

## Introduction

The prevention of diabetes and control of its micro- and macrovascular complications has become one of the main public health challenges for the 21^st^ century, with the aim of attaining a significant reduction in the huge premature morbidity and mortality it causes, with a parallel reduction in health care costs (Shaw et al. 2010). To this purpose, in addition to the implementation of strategies for diabetes prevention through lifestyle modification, a more aggressive control of the most important risk factors is urgently needed (American Diabetes Association 2009). In fact, despite strong evidence that pharmacological interventions play an important role in preventing complications in individuals with diabetes, the mere dissemination of evidence-based guidelines often fails to deeply influence clinical practice.

The considerable pressure on health care systems to provide high-quality care while controlling costs has led several public and private health care organizations to promote initiatives to measure and improve the quality of care for patients with diabetes (Peters et al. 1996; TRIAD Study Group 2002; Ilag et al. 2003; Saaddine et al. 2002,2006). Quality measures identified include process and intermediate outcome indicators, selected under the assumption that they are linked to downstream health outcomes. These measures have been widely utilized to monitor quality of care and promote continuous quality improvement initiatives (Saaddine et al. 2002,2006).

In Italy, the Associazione Medici Diabetologi (AMD) has implemented a continuous improvement effort involving over 300 diabetes clinics throughout the Country (Rossi et al. 2008). To this purpose, AMD identified a set of quality indicators, including process and intermediate outcome measures. All indicators are compared with reference values, or “gold standard”, established by identifying the best performers. By fixing as reference the results obtained by the best performers, specialists can easily realize the real margin of improvement made possible by simply increasing the level of attention to disease monitoring and treatment. The AMD Annals initiative allows a thorough description of the quality of care delivered every year by specialist structures to over 500.000 patients (Arcangeli et al. 2013). In additional to cumulative analyses, AMD Annals also provide sub-analyses at the regional level, to facilitate the local use of the data for quality improvement initiatives. The initiative has already proven to be effective in improving several quality of care indicators (Nicolucci et al. 2010).

Nevertheless, more structured benchmarking activities, involving discussion with clinicians, detection of obstacles at the local level, and identification of possible solutions are likely to produce further improvements in diabetes care. To this respect, a preliminary experience carried out in Sicily by a network of diabetes clinics documented a tangible improvement in the quality of diabetes care over 5 years (Club Diabete Sicili@ 2008).

In recent years, there has been an increasing recognition of patient-centered care as the best model to ensure a care respectful of, and responsive to patient preferences, needs, and values (Glasgow et al. 2008). The focus on patients’ perspective has thus become particularly relevant. Despite the consensus that patient-centered care is an essential component of good diabetes care, most diabetes performance measures are solely based on clinical measures, and do not include psychosocial items. The need to include psychosocial aspects in the quality model of diabetes care has been clearly emphasized by the international Diabetes Attitudes, Wishes, and Needs (DAWN) Program. The DAWN Study has clearly shown that people with diabetes across 13 countries have major psychosocial issues that are not adequately addressed in the existing health care systems (Conference Report 2004; International Diabetes Federation 2004; Peyrot et al. 2005a; Skovlund and Peyrot 2005; Wroe 2006). Given these premises, the BENCH-D Study (Benchmarking Network for Clinical and Humanistic Outcomes in Diabetes) was designed to test a model of regional benchmarking activity aimed at improving not only the quality of diabetes care, but also patient-centered outcomes. The BENCH-D study is integral part of the DAWN-2 initiatives (Peyrot et al. 2013) in Italy.

## Methods

### Objectives

Aims of the BENCH-D study are:

1. To tests the feasibility of a regional model for quality improvement, based on a deep analysis of regional data coupled with a structured process for the identification of priority actions;
2. To identify a core set of patient centered indicators to be adopted in conjunction with clinical indicators;
3. To evaluate the impact of the benchmarking initiative in improving both clinical and patient-centered quality indicators.

### Setting

The study involves 32 diabetes clinics in four different Italian regions (Piedmont, Marche, Lazio, and Sicily). Regions have been selected to reflect different levels of performance in terms of process and intermediate outcome measures, based on the information collected in the previous analyses of AMD Annals.

### Clinical data collection

Following the methodology applied in the AMD Annals initiative (Rossi et al. 2008; Arcangeli et al. 2013; Nicolucci et al. 2010), data are extracted from electronic databases of diabetes clinics using a specific software. Information on all patients seen in the clinic in the previous 12 months is collected, including age, gender, body mass index (BMI), smoking, diabetes duration, diabetes treatments, glycated hemoglobin (HbA1c), blood pressure and lipid profile values, microalbuminuria, serum creatinine, antihypertensive and lipid-lowering treatments, diabetes complications and other chronic conditions. A set of standard quality of care indicators is adopted, including process, intermediate and final outcomes, indicators of treatment intensity/appropriateness, and a global quality score.

Process measures are expressed as percentages of patients monitored at least once during the previous 12 months for the following parameters: HbA1c, blood pressure (BP), lipid profile, renal function, foot examination and eye examination.

Intermediate outcome measures include the proportion of patients with satisfactory values as well as the percentage of those with unacceptably high values. Satisfactory outcomes include HbA1c levels ≤7.0% (53 mmol/mol), BP values ≤130/80 mmHg, LDL cholesterol (LDL-C) levels <100 mg/dl, and body mass index (BMI) <27 Kg/m^2^. Unsatisfactory outcomes include HbA1c levels >8.0% (64 mmol/mol), blood pressure values ≥140/90 mmHg, LDL levels ≥130 mg/dl, BMI ≥30 Kg/m^2^, presence of micro/macroalbuminuria, and GFR ≤60 ml/min.

Indicators of treatment intensity/appropriateness take into consideration the use of pharmacological treatments in relation to the achievement of the targets. This set includes: no insulin treatment in spite of HbA1c > 9.0% (75 mmol/mol); HbA1c >9.0% (75 mmol/mol) in spite of insulin treatment; no lipid-lowering agents in spite of LDL-c ≥130 mg/dl; LDL-c ≥130 mg/dl in spite of lipid-lowering treatment; no antihypertensive treatments in spite of BP ≥ 140/90 mmHg; BP ≥ 140/90 mmHg in spite of antihypertensive treatment; no ACE-inhibitors (ACE-I) and/or Angiotensin Receptor Blockers (ARBs) in spite of micro/macroalbuminuria.

A quality of care summary score (Q score) is also calculated. The Q score has been developed and validated in two previous studies (De Berardis et al. 2008; Rossi et al. 2011); it is based on a combination of process and intermediate outcome indicators relative to HbA1c, blood pressure, LDL-cholesterol and microalbuminuria. The score ranges between 0 and 40; the higher the score, the better the quality of care. The risk to develop a new cardiovascular event within 3 years was shown to be 80% higher in patients with a score <15 and 20% higher in those with a score between 15 and 25, as compared to those with a score >25 (De Berardis et al. 2008; Rossi et al. 2011).

### Quality of life and patient satisfaction evaluation

Information on quality of life and patient satisfaction is collected using ad hoc questionnaires. The questionnaire includes ten instruments, partially derived from the DAWN-2 study (Peyrot et al. 2013).

Standardized instruments were incorporated into the questionnaires in original or shortened forms: SF-12 health survey, WHO Well-Being Index (WHO-5), Diabetes Empowerment Scale-Short Form (DES-SF), Problem Areas in Diabetes Scale 5 (PAID-5), Patient Assessment of Chronic Illness Care-Short Form (PACIC-SF), Health Care Climate Short Form (HCC-SF), Global Satisfaction with Diabetes Treatment (GSDT), Summary of Diabetes Self-Care Activities measure (SDSCA-6); Barriers to Medications (BM), Perceived Social Support (PSS).

All the questionnaires, with the exception of the already previously validated WHO-5 Well-Being Index (Bech et al. 1996; Hajos et al. 2013), have undergone a rigorous process of translation into the Italian language and psychometric validation specifically for the BENCH-D study. All the scale scores, with the exception of SDSCA-6, range between 0 and 100, with higher values indicating a higher level of the dimension measured.

- ***SF-12 Health Survey (SF-12)***: it is a widely used generic health status measure (Gandek et al. 1998). It includes 12 items which can be aggregated into two summary measures: the Physical (PCS) and Mental (MCS) Component Summary scores. Both scores are normalized to a general population mean of 50 and a standard deviation of 10.
- ***WHO-5 Well-Being Index (WHO-5)***: It assesses the psychological well-being, a core component of overall quality of life (Bech et al. 1996; Hajos et al. 2013). The use of WHO-5 is recommended in global and some national treatment guidelines for diabetes after its worldwide use in the DAWN initiative (Conference Report 2004; International Diabetes Federation 2004; Peyrot et al. [Bibr CR25][Bibr CR26]; Skovlund and Peyrot 2005; Wroe 2006). It is also a valid and reliable risk assessment measure for mild, moderate and severe depression and performs well in comparison to negative worded instruments such as the Brief Patient Health Questionnaire PHQ-9 or CES-D (Henkel et al. 2003; de Wit et al. 2007). It includes 5 items with responses on a six-point Likert scale. A score < 50 indicates poor psychological well-being, while a score ≤28 indicates likely depression.
- ***Diabetes Empowerment Scale – Short Form (DES-SF)***: DES-SF provides a very brief but well validated instrument to assess the patient’s confidence in taking an active role in own management of the condition (Anderson et al. 2000,2003). An effective diabetes management requires an empowered diabetes patient, who is able to take personal charge of parts of the care plan, yet the extent to which educational and pedagogic efforts of the healthcare team are effective in empowering the patient is rarely measured. The instrument includes 8 items with responses on a five-point Likert scale.
- ***Problem Areas in Diabetes – Short form (PAID-5)***: PAID evaluates the emotional diabetes distress, i.e. patients specific worries and negative emotions related to their diabetes (McGuire et al. 2010; Polonsky et al. 1995; Welch et al. 2003). It also complements the general well-being assessment with vital information about the patient’s acceptance of and coping with diabetes-specific issues. The instrument has been applied in more than a hundred studies and in the DAWN MIND (Monitoring Individual Needs in People with Diabetes) program across 10 countries (McGuire et al. 2010). The PAID-5 short-form has been validated in the multi-national DAWN MIND study and there is solid evidence that scores are clinically relevant. It includes 5 items with responses on a five-point Likert scale. A score ≥40 indicates high diabetes-related distress.
- ***Patients Assessment of Chronic Illness Care – Short form (PACIC-SF)***: In accordance with the evidence-based principles of patient-centered healthcare and the results of the DAWN initiative, the PACIC scale enables a concrete assessment of patient perceived access to support from the health care team according to a chronic care health delivery model (Glasgow et al. 2005; Gugiu et al. 2009). In this study we used the short-form version of the questionnaire, including 11 items with responses on a five-point Likert scale.
- ***Health Care Climate Questionnaire – Short form (HCC-SF)***: it evaluates the extent to which clinicians tend to favor the autonomy of the patient or, instead, tend to assume a paternalistic attitude towards the patient (Williams et al. 2004). This instrument has been used in many studies on different diseases, including diabetes, and has proven to be able to predict an improvement in metabolic control (Schafer et al. 1986). The study uses a short version of the questionnaire, consisting of 6 questions, with responses on 7-point Likert scale.
- ***Global Satisfaction with Diabetes Treatment (GSDT)***: It assesses the perceived impact of medication on daily life and psychological well being, in addition to overall satisfaction for the pharmacological treatment (Brod et al. 2006). It represents a very important element in determining the acceptance by the patient and adherence to therapy. The questionnaire was developed as part of the DAWN Study, and consists of 7 items with responses on 5-point Likert scales.
- **Summary of Diabetes Self-care Activities (SDSCA-6)**: it assesses health behaviours, notoriously difficult to evaluate reliably. The instrument has been shown to add relevant and useful information in the context of diabetes studies, even though data must be interpreted with caution as they are self-reported (Toobert et al. 2000). The version of the SDSCA-6 includes 6 selected items with responses on 8-point Likert scales, indicating the number of days (from 0 to 7) in a week the patient adheres with the recommended behaviour. Each item is treated individually.
- ***Barriers to Medications (BM)***: Compliance to medication is considered a key reason for suboptimal outcomes (Brod et al. 2006; Snoek et al. 2007; Anderson et al. 2004). BM is a simple questionnaire focused on assessing what concrete barriers patients face in daily life to taking their medication as scheduled. It has been developed in the DAWN study (Conference Report 2004; International Diabetes Federation 2004; Peyrot et al. [Bibr CR25][Bibr CR26]; Skovlund and Peyrot 2005; Wroe 2006) and includes 10 items with responses on 6-point Likert scales.
- ***Perceived social support (PSS)***: as demonstrated by the international DAWN study (Conference Report 2004; International Diabetes Federation 2004; Peyrot et al. [Bibr CR25][Bibr CR26]; Skovlund and Peyrot 2005; Wroe 2006), patient perception of access to support from multiple sources plays an important role. People with diabetes rely on support from the community, from family, peers and other sources on a daily basis, and understanding this level of support in addition to that provided by the healthcare system is essential, yet requires new questionnaires. The DAWN study included a very short questionnaire to assess satisfaction with support from various sources, which proved useful in determining the relative importance of these factors in patient’s self-management (Peyrot and International DAWN Youth Survey Group 2009). An optimized version of the questionnaire has been included in this study, including 5 items with responses on 5-point Likert scale.

Information on socio-demographic characteristics (age, gender, nationality, level of school education, working status, living status) are also collected through the questionnaire.

Questionnaires are anonymous, filled in by the patients on the occasion of a routine visit and sent to the coordinating center using sealed envelopes. The link between clinical and patient reported data is ensured by a univocal numerical code.

### Patient recruitment

All patients seen in the participating centers during the previous 12 months are included in the evaluation of clinical indicators. Patient centered outcomes are evaluated in a sub-sample of patients, selected using random sampling lists, stratified by diabetes treatment (oral agents, insulin, insulin and oral agents). An additional sample of patients with newly diagnosed diabetes (diagnosis made in the last 12 months) has been also identified. Within each treatment group, the sample has been further stratified by gender.

### Regional meetings

In each region, 5 meetings are scheduled, involving all the health care professionals participating in the initiative (diabetologists, nurses, dietitians).

In the first meeting, the aims and the operational aspects of the study were illustrated and discussed with participants.

The second meeting was focused on the discussion of the results relative to clinical indicators produced by the AMD Annals at the national and regional level. The “best performers” approach was used to compare the overall results obtained at the regional level with those obtained in those centers reaching the highest rates of performance for process and outcome measures. The session was organized according to a standardized structure. After the illustration of the results, each participant was asked to identify the three main problems emerging from the data. All the problems identified were then assigned by the participants a score from 0 to 10 for their relevance and a score from 0 to 10 for the concrete possibility to act on the problem. Problems were then ranked, and the first three were selected. Following the same procedure, three major obstacles causing the problems were identified and possible solutions proposed. The process led to the definition of a regional mandate, to be implemented in the following 12 months. The mandate did not require a radical change in the usual clinical practice, but rather an optimization of the work already done and a more appropriate use of the available resources.

The third meeting was focused on patient centered outcomes. Following the same methodology of the previous meeting, results of the quality of life and satisfaction survey were discussed, problems/barriers were identified, and possible solutions/actions proposed.

Eighteen months after the third meeting, clinical data will be extracted again, allowing a first evaluation of the impact of the initiative on clinical indicators. The impact of benchmarking activities on patient centered outcomes will be evaluated following a second survey conducted with the same patients participating in the first one. The results will be discussed on the occasion of the fourth meeting. Improvements registered and problematic areas where no improvements in performance will be documented will represent the core for discussion. Elements that have facilitated or represented an obstacle to improvement will be analyzed in detail, and actions to improve quality of care will be identified.

A final data collection on clinical indicators will be performed 12 months after the second clinical data extraction, and results will be discussed in the fifth meeting, which will represent the occasion for the assessment of the overall outcomes of the initiative. The results of this final assessment will be useful to identify the strengths and limitations of the approach adopted, and will help in better refining the overall approach before its extension to other regions.

The study design is reported in Table 1.

**Table 1:** **Study design**

Time	Period	Regional meeting	Activity
T-1	October - November 2010	First meeting	Presentation of the protocol
T0	November 2010 - July 2011		Administration of the first questionnaire to a random sample of patients. First clinical data extraction.
T2	September - October 2011	Second + third meeting	Discussion of clinical and patient-centered indicators and definition of the regional mandate.
T3	March 2012		Activation of regional mandate in the centers.
T4	March - July 2013		Patient recall and administration of the second questionnaire. Second clinical data extraction.
T5	September - November 2013	Fourth meeting	Intermediate evaluation of the impact of benchmarking on clinical indicators. Final evaluation of the impact of benchmarking on patient-centered indicators.
T6	July 2014		Third clinical data extraction.
T7	September - November 2014	Fifth meeting	Final evaluation of the impact of benchmarking on clinical indicators.

### Statistical analysis

Except for the SF-12 and the WHO-5, largely utilized in the Italian population, all the other quality of life scales have been translated, cross-culturally adapted and validated in Italian specifically for the BENCH-D study. The questionnaire was validated using a multitrait multi-item method (Ware et al. 1997). This method is used to determine whether each item in a scale is substantially related (r ≥0.40) to the total score computed from the other items in that scale (item convergent validity criterion). Internal-consistency reliability was estimated by the Cronbach’s alpha coefficient, with a minimum value of 0.70 considered as acceptable. Furthermore, the percentages of respondents achieving either the highest score (ceiling) and lowest score (floor) were calculated.

Patient characteristics and clinical indicators are described as mean and standard deviation or frequencies.

Scale scores are summarized as mean and standard deviation. All the data have been centrally analyzed anonymously.

### Ethics considerations

The study protocol was approved by local ethics committees of participating centers. A written informed consent was obtained by all patients before the administration of the questionnaires.

The protocol was approved by the institutional review board of the Mario Negri Sud Foundation and by the scientific committee of the Associazione Medici Diabetologi.

## Results

### Recruitment

Overall, 32 centers extracted by the electronic clinical record system information on 78,854 patients with type 2 diabetes for benchmarking activities based on clinical indicators, while 26 centers enrolled 2,390 patients for the validation and evaluation of patient centered outcomes. Socio-demographic and clinical characteristics of the patients who filled in the questionnaire are summarized in Table 2.

**Table 2:** **Socio-demographic and clinical characteristics of patients recruited in the BENCH-D study**

***Variable***		***Mean ± std or %***
**N**		2,390
**Socio-demographic characteristics**		
% Males		59.7%
Mean age (years)		65.0 ± 10.2
School education (%)	Primary school	39.2
	Middle school	28.8
	High school	25.4
	University	6.6
Working status (%)	Employed	23.5
	Housewife	14.4
	Retired	59.7
	Unemployed/student	2.4
Marital status (%)	Not married	6.8
	Married	77.9
	Separated/divorced	4.4
	Widower	10.9
Living status (%)	Alone	11.9
	Spouse/sons	82.8
	Other	5.2
Smokers (%)		16.5
**Clinical characteristics**		
Diabetes duration (yrs)		14.0 ± 15.3
HbA1c (%)		7.7 ± 1.5
HbA1c (mmol/mol)		61 ± 16.4
BMI (Kg/m^2^)		30.2 ± 6.0
Total cholesterol (mg/dl)		177.7 ± 39.9
HDL cholesterol (mg/dl)		49.5 ± 14.6
LDL cholesterol (mg/dl)		100.9 ± 32.7
Triglycerides (mg/dl)		142.4 ± 112.4
Systolic blood pressure		135.3 ± 16.2
Diastolic blood pressure		78.3 ± 9.1
Diabetes treatment (%)		
	Oral agents	49.5
	Oral agents + insulin	25.8
	Insulin	24.7
Lipid-lowering agents (%)		47.1
Antihypertensive treatment (%)		67.7
Diabetes complications	0	66.6
	1	25.0
	≥2	8.4

### Validation of the questionnaires

The validation process was performed using all the questionnaires filled in by 2,390 patients enrolled. All the questionnaires showed excellent psychometric characteristics; for all scales, the Cronbach’s alpha coefficient largely exceeded the minimum accepted value of 0.70 (Table 3). On the same line, item-scale correlation was extremely satisfactory for all the scales. Percentages at ceiling and floor were equal to 0% for DES-SF, HCC-SF, PACIC-SF, GSDT, PSS, and BM and in an acceptable range for PAID-5 (10.2% at floor and 0% at ceiling).

**Table 3:** **Quality of life and patient satisfaction questionnaires used and validated in the BENCH-D study**

Questionnaire	Abbreviation	Domain	No. of items	Scoring	Cronbach’s alpha	Item-scale correlation (Min-Max)
SF-12 Health Survey physical component	SF-12 PCS	Physical functioning	6	0-100	-	-
WHO-5 Well-Being Index	WHO-5	Psychological well-being	5	0-100	-	-
Diabetes Empowerment Scale-Short Form	DES-SF	Diabetes Psychosocial Self-Efficacy	8	0-100	0.93	0.916-0.933
Problem Areas in Diabetes 5	PAID-5	Diabetes distress	5	100-0	0.93	0.918-0.923
Health Care Climate Questionnaire-Short Form	HCC-SF	Patient centered communication	6	0-100	0.93	0.909-0.927
Patients Assessment of Chronic Illness Care-Short Form	PACIC-SF	Quality of chronic illness care and patient support	11	0-100	0.85	0.833-0.849
Global Satisfaction for Diabetes Treatment	GSDT	Satisfaction with treatment regimen	7	0-100	0.80	0.748-0.787
Diabetes Self-care Activities	SDSCA-6	Self-care activities	6	0-7	-	-
Barriers to Medications	BM	Barriers to medication	10	0-100	0.77	0.736-0.753
Perceived social support	PSS	Patient perceived support	5	0-100	0.81	0.748-0.830

### Regional meetings

Meetings for the structured discussion of clinical and humanistic indicators have been held between September and October 2011. The approach adopted led to the identification of different priorities for intervention in the four regions, reflecting different organizational characteristics and specific needs.

The operational protocol has been officially launched in March 2012. The biggest challenge identified in all regions was to test innovative ways of communication with the patient, and to deal with his/her psychosocial needs. Table 4 shows as an example the problems and solutions proposed in one of the regions.

**Table 4:** **Examples of actions identified in the regional mandate**

Problem area	Action	Quality indicator to measure impact of benchmarking
Many patients with microalbuminuria are not treated with ACE-inhibitors and/or ARBs	Use of an ad hoc query on the electronic clinical record to identify these patients	Reduction in the percentage of patients with microalbuminuria not treated with ACE-inhibitors and/or ARBs
Many patients with LDL-cholesterol ≥130 mg/dl are not treated with lipid-lowering agents	Use of an ad hoc query on the electronic clinical record to identify these patients	Reduction in the percentage of patients with LDL-C ≥130 mg/dl not treated with lipid-lowering agents
Poor proactive involvement of patients in the process of care	Identification of a model of “therapeutic contract”, with discussion and agreement on therapeutic goals and appointments for regular monitoring of diabetes complications	Increase in the percentage of patients reaching HbA1c, blood pressure and LDL-cholesterol targets. Increase in percentage patients monitored for nephropathy, retinopathy and diabetic foot
Poor attention to patient with low levels of psychological well-being	Use of WHO-5 to identify patients with score <28; referral for specific counseling, identification of barriers and solutions	Increase in mean values of WHO-5 and DES-SF scores and decrease in mean values of PAID-5

## Discussion

The BENCH-D study allows the field testing of benchmarking activities based on a structured discussion of quality indicators, selection of priorities, and identification pertinent actions. Furthermore, the study includes for the first time in a benchmarking initiative a set of validated patient centered indicators and will document whether it is possible to improve not only clinical indicators, but also patient well-being and satisfaction with the care received. If successful, the initiative could be extended to other Italian regions, as well as to other health care systems. As a whole, this project can make an important contribution in promoting patient-centered care and improving the quality of diabetes care.

## Abbreviations

AMD: Associazione Medici Diabetologi
DAWN: Diabetes Attitudes, Wishes, and Needs (DAWN) Program
BENCH-D: Benchmarking Network for Clinical and Humanistic Outcomes in Diabetes Study
BMI: Body mass index
HbA1c: Glycated hemoglobin
BP: Blood pressure
LDL-c: LDL cholesterol
ACE-I: ACE-inhibitors
ARBs: Angiotensin Receptor Blockers
Q score: Quality of care summary score
SF-12 PCS: SF-12 Health Survey physical component
WHO-5: WHO-5 well-being index
DES-SF: Diabetes Empowerment Scale-Short Form
PAID-5: Problem Areas in Diabetes 5
HCC-SF: Health Care Climate Questionnaire-Short Form
PACIC-SF: Patients Assessment of Chronic Illness Care-Short Form
GSDT: Global Satisfaction for Diabetes Treatment
SDSCA-6: Diabetes Self-care Activities
BM: Barriers to Medications
PSS: Perceived social support.
